# A Support Vector Machine-Assisted Metabolomics Approach for Non-Targeted Screening of Multi-Class Pesticides and Veterinary Drugs in Maize

**DOI:** 10.3390/molecules29133026

**Published:** 2024-06-26

**Authors:** Weifeng Xue, Fang Li, Xuemei Li, Ying Liu

**Affiliations:** Technology Centre of Dalian Customs, Dalian 116000, China; fanglee_4125@163.com (F.L.); lxmciq@163.com (X.L.)

**Keywords:** maize, marker compounds, metabolomics, non-targeted screening, pesticides and veterinary drugs, support vector machine

## Abstract

The contamination risks of plant-derived foods due to the co-existence of pesticides and veterinary drugs (P&VDs) have not been fully understood. With an increasing number of unexpected P&VDs illegally added to foods, it is essential to develop a non-targeted screening method for P&VDs for their comprehensive risk assessment. In this study, a modified support vector machine (SVM)-assisted metabolomics approach by screening eligible variables to represent marker compounds of 124 multi-class P&VDs in maize was developed based on the results of high-performance liquid chromatography–tandem mass spectrometry. Principal component analysis and orthogonal partial least squares discriminant analysis indicate the existence of variables with obvious inter-group differences, which were further investigated by S-plot plots, permutation tests, and variable importance in projection to obtain eligible variables. Meanwhile, SVM recursive feature elimination under the radial basis function was employed to obtain the weight-squared values of all the variables ranging from large to small for the screening of eligible variables as well. Pairwise *t*-tests and fold changes of concentration were further employed to confirm these eligible variables to represent marker compounds. The results indicate that 120 out of 124 P&VDs can be identified by the SVM-assisted metabolomics method, while only 109 P&VDs can be found by the metabolomics method alone, implying that SVM can promote the screening accuracy of the metabolomics method. In addition, the method’s practicability was validated by the real contaminated maize samples, which provide a bright application prospect in non-targeted screening of contaminants. The limits of detection for 120 P&VDs in maize samples were calculated to be 0.3~1.5 µg/kg.

## 1. Introduction

It has always been a headache to resolve pesticide contamination in plant-derived foods, which poses a considerable threat to global food safety. Some national governments (e.g., China [1], the United States [2], and Japan [3]) and international authorities (e.g., the European Union [4]) have issued a series of formal regulatory documents on the maximum residue limits (MRLs) of pesticides in plant-derived foods. With the deepening of research, more and more evidence has shown that plant-derived foods are also suffering from serious contamination by veterinary drugs [5,6,7,8,9,10,11,12,13]. Previous studies [5,6,7,8,9,10,11,12,13] have shown that veterinary drugs excreted by livestock and poultry are commonly used as fertilizers on farmland, and some plant-derived foods (e.g., maize, potatoes, cucumbers, and lettuce) are easy to absorb from the soil, resulting in a variety of veterinary drugs (e.g., tetracycline, quinolones, and sulfonamides) accumulating in the foods, with a total concentration up to several mg/kg levels [8,14,15,16,17]. Due to the lack of risk evaluation standards for veterinary drugs in plant-derived foods at home and abroad, it is difficult to directly judge whether the concentration of veterinary drugs will cause adverse effects. Referring to the regulatory documents of MRLs of veterinary drugs in animal-derived foods [18,19], the concentration of 10 μg/kg was deemed the safety threshold for most veterinary drugs, and it is inferred that the reported concentration level of veterinary drugs in some plant-derived foods is likely to cause serious food safety incidents. For example, some organophosphorus pesticides can damage the nervous system, while sulfonamide antibiotics can cause allergic reactions after entering the human body. To sum up, resolving the food safety issue caused by the co-existence of pesticides and veterinary drugs (P&VDs) is urgent, and developing an effective screening method is a feasible way to prevent the occurrence of potential contamination risks. 

Traditional methods to screen contaminants in foods are mostly based on the establishment of a set of databases containing a variety of P&VDs [20,21,22]. These methods have proved to be successful in discerning contaminants in the database, but they are helpless for contaminant identification outside the database, which casts a shadow on food safety. For example, some infamous food safety incidents took place in the 21st century, including melamine milk powder and fipronil eggs, which arose from some enterprises or individuals illegally adding unexpected contaminants not in the routine test scope of foods. Even if the concentrations of these contaminants were highly above the red line of food safety, these defective products were also considered to be qualified. As a result, they have gradually developed into extremely serious food safety incidents. It can be seen that the constant establishment of new databases of contaminants to screen unexpected P&VDs in plant-derived foods is a passive way that consumes a lot of human and material resources and is also unable to meet the increasing demands for the detection of unexpected P&VDs in foods. Therefore, it has become a popular trend to develop highly efficient non-targeted screening methods for contaminants, as the European Commission NORMAN network proposed [23,24]. 

Metabolomics has shown great promise in screening unexpected contaminants in the food safety field due to its advantages in handling massive data with complicated characteristics, such as small sample sizes, mass interferents, and high noise [25,26,27,28,29]. Marker compounds on behalf of unexpected contaminants in foods were screened and identified by in-house or network databases (e.g., Massbank, SciFinder, ChemSpider, PubChem, and Metlin) during metabolomics analysis in order to achieve non-targeted screening of contaminants [28,29]. 

As we know, metabolomics data are composed of fewer samples but massive features (i.e., variables). How to extract meaningful information and find the eligible variables on behalf of marker compounds is a primary issue for researchers to resolve. Generally speaking, the distinguishable factors for metabolomics data between any two groups (e.g., experiment vs. control) are a combination of variables rather than a single one. Therefore, several chemometric approaches, such as principal component analysis (PCA) [30,31,32], orthogonal partial least squares discriminant analysis (OPLS-DA) [30,31,32], random forest (RF) [33,34], and support vector machine (SVM) [33,35,36], have widely been used to deal with these metabolomics data. Among all these modeling methods, SVM is gaining popularity in a wide variety of metabolomics studies due to its prediction performance. SVM is known to have excellent generalization ability and can be applied to nonlinear cases with the assistance of kernels relative to PCA and OPLS-DA, with only an assumption of linearity. SVM recursive feature elimination (SVMRFE) is a wrapper approach that adopts the rule of weight (*w*) to rank the variables and works well when the number of samples is small but the number of variables is large. Due to this feature of SVMRFE, which is similar to that of metabolomics data, SVMRFE is often used in metabolomics studies [37,38,39,40]. In addition, SVMRFE has shown better predictability than OPLS-DA. To date, SVMRFE-involved metabolomics studies are mainly applied to seek eligible biomarkers for disease diagnosis, but they remain insufficient to complete the non-targeted screening of contaminants in foods. In this work, we try to apply SVM to metabolomics analysis to better screen and identify marker compounds on behalf of 124 multi-class P&VDs in maize so as to realize non-targeted screening of residual contaminants in plant-derived foods.

## 2. Results and Discussion

### 2.1. Data Preprocessing

**Figure 1:**
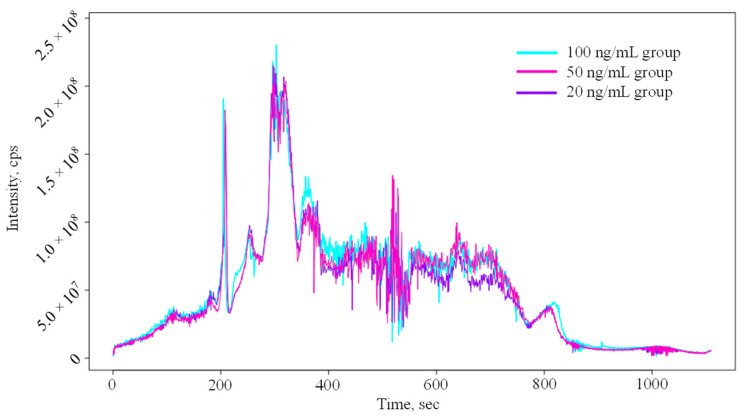
Total ion chromatograms of spiked maize sample groups on the W4M platform.

The total ion chromatograms containing 124 P&VDs in three concentration groups are shown in Figure 1. The matrix complexity of maize may cause the recoveries of each contaminant in different samples to be significantly discrepant. As a result, no evident correlation between peak intensities and concentration levels was observed, as shown in Figure 1. To eliminate the peak intensity errors of variables arising from different recoveries of P&VDs during the pretreatment process, enrofloxacin-d5 (parent ion *m*/*z* 365.21092; fragment ions *m*/*z* 348.19692, 322.21804, and 246.11012; retention time 7.01 min) and atrazine-d5 (parent ion *m*/*z* 221.14017; fragment ions *m*/*z* 179.08602, 137.06393, and 101.08703; retention time 5.77 min) were jointly spiked for recovery calibration. Blank maize extract solutions were employed to prepare the standard curves (5, 10, 25, 50, and 100 ng/mL) of enrofloxacin-d5 and atrazine-d5 to calculate the recoveries of these two deuterated compounds in maize samples, with the recoveries to be 75.6%~93.1%, 72.9%~93.4%, and 74.9%~93.6% for enrofloxacin-d5 and 73.6%~95.2%, 81.2%~94.7%, and 72.9%~96.4% for atrazine-d5 in the 20, 50, and 100 ng/mL groups, respectively (Table S1, Supplementary Materials). 

For each sample, we adopted the formula of 2 × 100%/((100%/recovery of enrofloxacin-d5) + (100%/recovery of atrazine-d5)) to calculate the final recovery of internal standards (Table S1), whose average value from the same concentration group needs to multiply a calibration coefficient to obtain 100% recovery. Peak intensities for internal standards and all other variables were also calibrated after multiplying the coefficient. After this, eligible variables with a relative standard deviation of peak intensity less than 30% in QC and three concentration groups were obtained to establish a 1447 × 39 data matrix for further analysis [41].

### 2.2. Multivariate Analysis

#### 2.2.1. PCA Results

**Figure 2:**
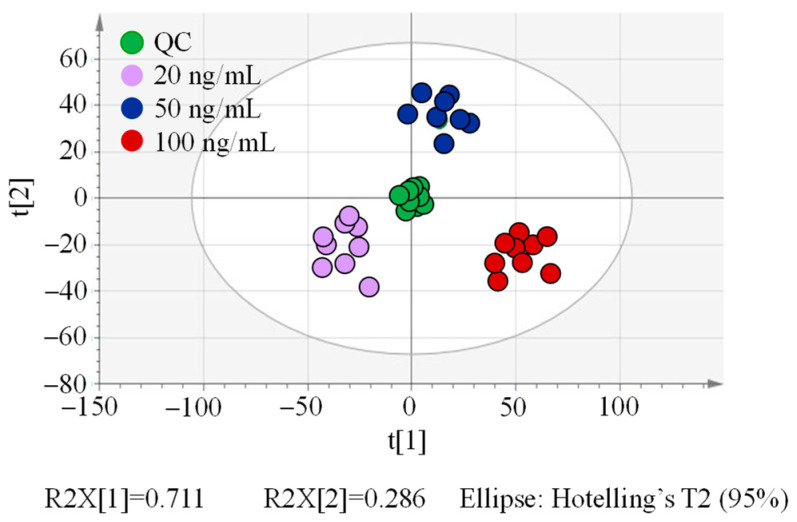
PCA score plot of spiked maize sample groups.

As indicated in Figure 2, all samples showed their credibility within a 95% confidence level. QC group samples have a close gathering, implying that the high quality of the data was available and deserved further analysis [42]. Nine samples from the same concentration group clustered closely, indicating the good intra-group similarity of these samples. Inter-group samples separated obviously on the first principal component axis, implying distinguishable differences in variables existed among these groups, which provided the possibility to hunt for marker compounds among concentration groups.

#### 2.2.2. Cluster Analysis Results

Cluster analysis can directly describe the similarity level of samples, meaning that samples with the highest similarity gather together preferentially, level by level, until all the samples finish their aggregation. As observed in Figure S1 (Supplementary Materials), the samples from the same concentration group were obviously separated from others, showing their high intra-group congeniality. The evident inter-group separation can be attributed to the differences in peak intensities of variables. The findings of cluster analysis were consistent with those of PCA, and both supported the existence of variables with significant differences.

#### 2.2.3. OPLS-DA Results

**Figure 3:**
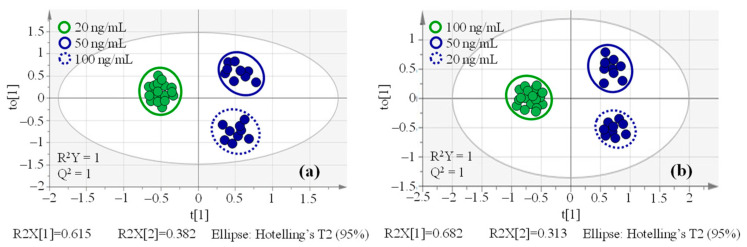
OPLS-DA score plots of spiked maize sample groups ((**a**): 20 ng/mL vs. 50 & 100 ng/mL; (**b**): 100 ng/mL vs. 20 & 50 ng/mL).

As we know, OPLS-DA is a two-class classification model; the size imbalance of two classes may introduce a bias in the computation of the decision rule, which can result in the unreliability of the OPLS-DA model [43]. To resolve this issue, we adopted the synthetic minority over-sampling technique (SMOTE) [44] to increase the sample size of the class with fewer samples. Herein, when the 20 ng/mL concentration group was designed as Class 1 (only 9 samples), and the 50 and 100 ng/mL concentration groups as a whole (18 samples in total) were designated Class 2, it needed to increase the size of Class 1 from 9 to 18 samples (Figure 3a). Similarly, the 100 ng/mL concentration group in Class 1 also needed to increase the class size to match that of the 20 and 50 ng/mL concentration groups in Class 2 (Figure 3b). As indicated in Figure 3, the obvious separation between Class 1 and Class 2 on the first principal component axis in OPLS-DA score plots was observed, implying that the differences in variables were significant between the two classes. R^2^Y and Q^2^ are two key parameters in the OPLS-DA model to evaluate the interpretability along the Y-axis and the predictability of the model, respectively [45,46]. When R^2^Y and Q^2^ values are close to 1, it means that the OPLS-DA model has high reliability and predictability. In Figure 3, the R^2^Y and Q^2^ values were both equal to 1, which favored the robustness of these OPLS-DA models. 

**Figure 4:**
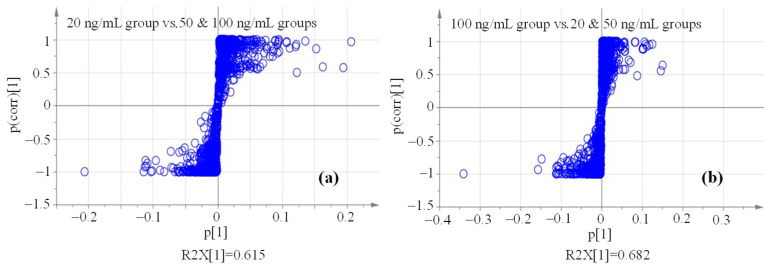
S-plot plots of spiked maize sample groups ((**a**): 20 ng/mL vs. 50 & 100 ng/mL; (**b**): 100 ng/mL vs. 20 & 50 ng/mL).

A series of data points to represent the variables were shown in the S-plot plots (Figure 4), in which the points near the two tips of the ‘S’ plots signified the larger contribution and higher confidence levels of the corresponding variables in differentiating two classes of the OPLS-DA model. These variables were qualified to be marker compound candidates. Herein, marker compounds in the significantly low (20 ng/mL) and high (100 ng/mL) concentration groups should be sought at the right end of Figure 4a and the left end of Figure 4b, respectively.

**Figure 5:**
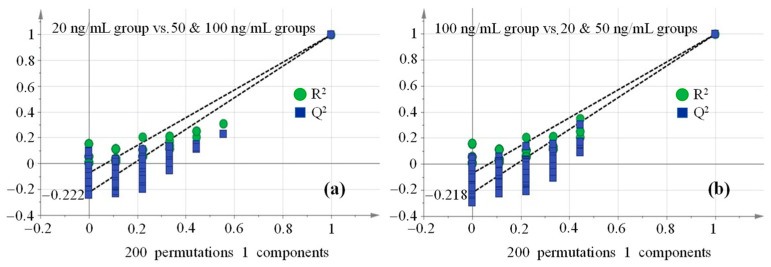
Permutation test plots of spiked maize sample groups ((**a**): 20 ng/mL vs. 50 & 100 ng/mL; (**b**): 100 ng/mL vs. 20 & 50 ng/mL).

As we know, the Y-axis in the permutation test plot is on behalf of R^2^Y and Q^2^Y for the model, and the X-axis represents the correlation coefficient between original and permuted response data. It is desirable to summarize the results of the permutation test in a quantitative manner. One way to do this is to conduct conventional regression analysis on the two sets of points, i.e., one regression line is fitted among the R^2^Y points (green circles) and another line among the Q^2^Y points (blue squares), as shown in Figure 5. The intercepts of the resulting regression lines are interpretable as measures of ‘background’ R^2^Y and Q^2^Y obtained by fit to random data. Experience shows that the R^2^Y-intercept should not exceed 0.3~0.4 and that the O^2^Y-intercept should not exceed 0.05. Intercepts below these limits indicate valid models. A 200-iteration permutation test was a common method to evaluate whether the OPLS-DA model underwent over-fitting by the significance test (*p*-value) of Q^2^Y metrics [47]. If *p* < 0.05, it meant that the OPLS-DA models were free of over-fitting, as reflected by the Q^2^Y-intercept being less than 0.05 [47]. Figure 5 clearly points out that all OPLS-DA models were immune to over-fitting under the principles mentioned above.

VIP, as a measure of the importance of the variable in the OPLS-DA model, was computed for each extracted variable. Usually, VIP > 1 was considered to be the crucial threshold to pick eligible variables on behalf of marker compounds. In this study, a total of 228 variables with VIP > 1 from 20 concentration groups vs. 50 and 100 concentration groups (20 vs. 50 and 100 in abbreviation applied below) were chosen to be the marker compound candidates, but only 188 variables were eligible from 100 concentration groups vs. 20 and 50 concentration groups (100 vs. 20 and 50 in abbreviation applied below). One hundred and thirty-nine variables were observed to be overlapped between two VIP lists.

#### 2.2.4. SVM Results

After running SVM codes, all the classification accuracy of the 10-fold cross-validation was computed to be 100%, in favor of the models with good generalization ability. The best penalty factor (c) and best kernel function parameter (g) from 3-fold cross-validation at 100% accuracy rate were computed to be 0.0039 and 0.0039 for 20 vs. 50 and 100, and 0.0037 and 0.0037 for 100 vs. 20 and 50. Two hundred and twenty-eight variables with VIP > 1 obtained from 20 vs. 50 and 100 were included in the weight table of two hundred and fifty-three variables. Similarly, 188 variables with VIP > 1 corresponded to those in the weight table, which contained an additional 26 variables with VIP < 1 from 100 vs. 20 and 50. From the above, we can see that the overlap ratio of variables obtained between VIP and weight ranking methods was 90.1% (i.e., 228 × 100%/253) from 20 vs. 50 and 100, while this ratio fell to 87.8% (i.e., 188 × 100%/214) from 100 vs. 20 and 50, indicating that the variables sought by the two methods were of high consistency, which laid a solid foundation to accurately seek eligible variables to represent marker compounds. One hundred and fifty-four overlapped variables from the two weight lists mentioned above were obtained for univariate analysis to further confirm the validity of those variables.

### 2.3. Univariate Analysis

Pairwise *t*-tests [47,48,49] and fold changes of concentration [27,50] have proved to be appropriate for univariate analysis of the metabolomics data. A pairwise *t*-test focuses on the significant differences of variables in peak intensity between two concentration groups, while a fold change of concentration considers the peak intensity ratio of variables between the high and low concentration groups. In general, the variables with a significance level (*p*) of pairwise *t*-tests below 0.05 and fold change (FC) of concentration above 2 are deemed eligible. In this study, *p* values of 154 variables between 20 and 50, 20 and 100, as well as 50 and 100 ng/mL concentration groups, i.e., *p*_20vs.50_, *p*_20vs.100,_ and *p*_50vs.100_, were calculated to be lower than 0.05 in a pairwise *t*-test (Table S2), indicating that the significant inter-group differences of 154 variables indeed existed. In addition, the fold changes of concentration between 50 and 20 (FC_50vs.20_), as well as 100 and 20 ng/mL groups (FC_100vs.20_), were also calculated to be 2.333~2.671 and 4.555~5.332 (Table S3), which were above 2 to further support the eligibility of 154 variables as marker compound candidates.

As shown in Table 1, 120 out of 154 variables were confirmed as marker compounds (124 in total) by the weight ranking method, with a screening rate of over 96% (i.e., 120 × 100%/124), while the VIP method can only find 109 marker compounds with a screening rate of about 88% (i.e., 109 × 100%/124), showing that the weight ranking method expressed better performance in selecting eligible variables to represent marker compounds than the VIP method. Fleroxacin, lincomycin, clindamycin, and mebendazole, as four veterinary drugs, have no variables equal to their identities as marker compounds in weight and VIP lists. To be different with these four contaminants, three pesticides (i.e., pyridaben, hexazinone, and phosmet) and another eight veterinary drugs, including sulfaphenazole, sulfamethoxazole, sulfamethoxypyridazine, lomefloxacin, fenthion, danofloxacin, sparfloxacin, and tilmicosin, played the role of unique marker compounds, which corresponded to the variables with VIP < 1 from 20 vs. 50 and 100, but were also included in the weight lists (Table 1). Thirty-four in one hundred and fifty-four variables not identified as marker compounds in weight lists showed more complicated and erratic LC–MS/MS and metabolomics information, which needed more in-depth studies to discern their origins from a matrix or foreign impurity.

A blank maize sample was also investigated by the SVM-assisted metabolomics method with no corresponding variables of 124 P&VDs found, eliminating the inherent (rather than spiked) interference of these contaminants in maize to screen marker compounds and the existence of false positives.

### 2.4. Limits of Detection

According to the method proposed by the US Environmental Protection Agency to calculate the limits of detection (LODs) of 120 P&VDs [51], we first prepared a 2.0 g blank maize sample for the same pretreatment as introduced above to obtain a 1 mL extract solution. In this extract, we spiked a mixed solution (1 µg/mL, 20 µL) containing 120 P&VDs, resulting in a final concentration of 20 ng/mL. This process was repeated to gain seven replicates, which were also subjected to metabolomics analysis to obtain peak intensities of 120 P&VDs. For each P&VD, the concentration level of 20 ng/mL corresponds to the peak intensity average value of seven replicates. Therefore, the concentration (ng/mL) of each P&VD can be calculated by the formula for the respective peak intensity × 20/average peak intensity. As shown in Table 1, the LOD results for 120 P&VDs varied between 0.3 and 1.5 µg/kg.

The metabolomics method has successfully been applied to non-targeted contaminant screening in plant-derived foods such as tea [27], lettuce [29], maize [29], and orange juice [33]. In contrast to these studies, which only focused on pesticides or veterinary drugs, our study expands the domain of target compounds to simultaneously screen multi-class P&VDs. This is the first time to prove the introduction of SVM as an effective tool for promoting the screening rate of the metabolomics approach. In addition, method LODs were calculated to be significantly below 10 µg/kg for 120 P&VDs, which even have the upper hand over some targeted multi-residue methods [20,21,22].

### 2.5. Practicability Test

For plant-derived foods, the contamination pathways of pesticides are almost explicit; however, the contamination sources of veterinary drugs have not been fully understood. As reported in previous studies [52,53,54], the application of fertilizer and irrigation water contaminated with veterinary drugs onto farmland is a primary reason to cause the contamination of plant-derived foods; we hypothesized that some plant-derived foods from rural areas exposed to the abuse of veterinary drugs in livestock farming may suffer higher contamination risks. To investigate the practicability of our proposed SVM-assisted metabolomics screening method, we adopted maize samples from Qili and Bali villages as a case study. These two villages in Jinpu New Area, affiliated with Dalian City, are not only crucial maize suppliers but also pivotal providers of fresh livestock and poultry meat, which makes the maize samples highly susceptible to contamination by veterinary drugs.

Maize samples were collected after the maturity period in late September 2023. After metabolomics analysis, four contaminants, including norfloxacin, enrofloxacin, imidacloprid, and carbendazim, were all detected in two villages, with their concentrations being 10.7~17.7 µg/kg (Table S4). The abundance of norfloxacin and enrofloxacin in maize samples can be attributed to their extensive use as medicines and feed additives in animal husbandry [55], while the residual imidacloprid and carbendazim in maize samples with relatively high concentrations can be due to their widespread application to combat aphid [56] and bacterial wilt [56], respectively. As indicated in Table S4, the contamination of pesticides remained predominant over that of veterinary drugs, and imidacloprid presented the highest concentration among the four contaminants in both villages. In addition, Qili village suffered more serious overall contamination from four contaminants than Bali village. The test results supported the practicability of our proposed SVM-assisted metabolomics method in non-targeted screening of contaminants. It is worth noting that all four contaminants identified by the method presented a concentration above 10 µg/kg, which was understandable to show the feasibility of the method. But for those P&VDs with concentrations below 10 µg/kg, it is still unclear how to prove the applicability of the method, which probably led to those contaminants being unable to be detected. That is to say, the actual contamination of maize by P&VDs in the Jinpu New Area can be even worse than what we expected, and it deserves more attention to defuse the underlying risks. Food safety has no borders. In view of some food issues that initially occurred in local areas on a small scale, it is still essential for any country and international organization to come to notice and to strengthen cooperation in the prevention and control of the issues, such as through information sharing, experience exchange, and even the publication of standardized documents to ensure food safety from the source.

## 3. Materials and Methods

### 3.1. Chemicals and Materials

Maize samples were purchased from a local farmer’s market in Dalian City, China. Methanol and acetonitrile (HPLC grade) were purchased from Merck Corporation (Darmstadt, Germany). Formic acid and acetic acid (HPLC grade) were provided by China Shanghai ANPEL Laboratory Technologies Inc. (Shanghai, China). Ammonium formate, anhydrous sodium sulfate (Na_2_SO_4_), anhydrous sodium acetate (NaAC), and primary secondary amine (PSA) sorbent were obtained from China Sinopharm Chemical Reagent Co., Ltd. (Shanghai, China). Filter membrane (0.22 µm) was purchased from Agilent Technologies (Santa Clara, CA, USA). Ultrapure water was produced by the Milli-Q ultrapure water system from Merck Corporation (Darmstadt, Germany). Enrofloxacin-d5 and atrazine-d5 (100 μg/mL in methanol) for recovery calibration were provided by First Standard (Worcester, MA, USA). Sixty-nine pesticides and fifty-five veterinary drugs were purchased from the corporations First Standard (Worcester, MA, USA), Sigma (St. Louis, MO, USA), Dr. Ehrenstorfer (Wesel, Germany), and TRC (Montréal, QC, Canada), with a purity greater than 98%. The detailed information on these P&VDs is shown in Table 2.

### 3.2. Solution Preparation

A total of 124 P&VDs was separately prepared in methanol with a concentration of 100 µg/mL as the stock solution, 1 mL of which was taken, mixed together, and diluted with methanol to prepare a 1 µg/mL working solution. A 100 ng/mL mixed working solution of enrofloxacin-d5 and atrazine-d5 was prepared by diluting their 100 µg/mL stock solution with methanol.

### 3.3. Sample Preparation and Pretreatment Process

(a) The maize sample was ground into powder by a grinder; (b) 2.0, 5.0, and 10.0 g of maize powder, together with corresponding 20, 50, and 100 µL of 124 P&VDs mixed solutions (1 µg/mL), were poured into 50 mL polypropylene centrifuge tubes, respectively. To calibrate the recovery during the sample pretreatment process, a mixed solution (0.5 mL, 100 ng/mL) of enrofloxacin-d5 and atrazine-d5 was further added as recovery internal standards; (c) a total of 20 mL of acetonitrile/water (80/20, *v*/*v*) with 1% acetic acid was dumped into the tube by vortex and ultrasonic extraction for 1 and 30 min, respectively. A total of 6.0 g anhydrous Na_2_SO_4_ and 1.5 g NaAC were added, shocking violently for 1 min and centrifuging at 6000 r/min for 5 min; (d) all the supernatant solutions were taken into a new tube containing 2.0 g anhydrous Na_2_SO_4_ and 0.3 g PSA, centrifuging at 6000 r/min for 2 min; (e) all the solutions were extracted, dried with nitrogen gas flow, and redissolved with 1 mL 40% (*v*/*v*) methanol–4 mmol/L ammonium formate buffer solution, vortexing for 1 min; (f) filtered with a 0.22 µm filter membrane, the sample solutions of 124 P&VDs at the theoretical concentrations of 20, 50, and 100 ng/mL were prepared. Each concentration experiment was completed in nonuplicate to obtain replicate samples.

### 3.4. Sample Grouping and Naming

Twenty-seven samples named 20 ng/mL−1~20 ng/mL−9, 50 ng/mL−1~50 ng/mL−9, and 100 ng/mL−1~100 ng/mL−9 were obtained from three concentration groups. A quality control (QC) sample was prepared by mixing 30 µL of each sample from the above-mentioned concentration groups [27,57,58] and underwent three repeated injections into the LC–MS/MS system before and after the injection of each concentration group. Twelve injections of QC samples were named after QC-1, QC-2, …, and QC-12 to evaluate the stability of LC–MS/MS.

### 3.5. Analytical Method

A quadrupole/electrostatic field orbitrap LC–MS/MS system (Q Exactive Plus, Thermo Fisher Scientific Inc., Waltham, MA, USA) equipped with a heatable electrospray ion (ESI) source was used to analyze all pesticides, veterinary drugs, enrofloxacin-d5, and atrazine-d5. The ESI-positive mode was adopted to obtain M+H adducts for metabolomics analysis. An Accucore RP-MS column (100 mm × 2.1 mm, 2.6 µm particle diameter) purchased from Thermo Fisher Corporation (Waltham, MA, USA) was employed to separate components after an injection of 10 µL sample solution. The mobile phases were composed of 0.1% (*v*/*v*) formic acid and ammonium formate at 4 mmol/L in water (eluent A) and 0.1% (*v*/*v*) formic acid and ammonium formate at 4 mmol/L in methanol (eluent B). The flow rate was kept at 0.3 mL/min. The elution program was as follows: 100% A (0 min), 100% A (2 min), 0% A (8 min), 0% A (13 min), and 100% A (14 min) until the end of the run. The oven temperature was kept at 40 °C. The Q Exactive Plus parameter settings include (a) heating and capillary temperature at 320 °C; (b) sheath and auxiliary gas from N_2_, with flow rates of 40 and 10 arb, respectively; (c) lens and spray voltage at 50 and 3200 V, respectively; (d) scan mode to be full-scan/data-dependent two-stage scanning; (e) MS parameters to be full-scan resolution at 70,000, AGC target at 1 × 10^6^, maximum dwell time at 100 ms, and scan range of *m*/*z* between 100 and 1000; (f) MS/MS parameters to be resolution at 17,500, AGC target at 2 × 10^5^, and maximum dwell time at 50 ms.

Trace Finder software (Version 3.3) installed on the LC–MS/MS system was employed for residue analysis, with specific screening conditions as follows: (a) for primary parent ions, the response intensity threshold at 10,000, the ratio of signal to noise at 5.0, and mass error at 5 ppm; (b) for secondary fragment ions, the minimum number of matching ions at 1, the response intensity threshold at 10,000, and mass error at 5 ppm. The quantification of enrofloxacin-d5 and atrazine-d5 was finished by standard curves on the basis of the peak areas of primary parent ions for recovery calibration.

### 3.6. Metabolomics Data Processing

LC–MS/MS output files cannot be directly employed for metabolomics analysis and need to be converted from .RAW format to .mzXML format [59]. The new-formatted files were uploaded onto the Workflow4Metabolomics (W4M) platform (https://workflow4metabolomics.usegalaxy.fr/, accessed on 19 October 2022) for further analysis [60]. Chromatographic peak detection, alignment, and retention time calibration were performed on the platform, with further operations including normalization, centralizing, scaling, and data transformation of peak intensity to acquire the data matrix, in which variable and sample names were designated abscissa and ordinate, respectively [60,61]. PCA [62,63,64] and OPLS-DA [65,66] embedded in SIMCA software (Version 14.1) [67], together with cluster analysis in Heml software (Version 1.0.3.7), were performed for multivariate analysis of the data matrix. Over-fitting of the OPLS-DA model was evaluated by permutation tests [67,68]. Variable importance in projection (VIP) > 1 is the threshold to screen variables as marker compound candidates [67,68,69]. Under this principle, eligible variables from three concentration groups were included in the VIP lists.

SVM can perform nonlinear classification by mapping the input data into a high-dimensional feature space, the process of which is known to be the kernel trick [70]. Many kernel functions are available, but the most commonly used one is the radial basis function (RBF). Therefore, we chose SVMRFE under RBF to run the program in MATLAB software (Version R2019b). A 10-fold cross-validation was performed by randomly dividing the dataset into 10 subsets and computing the mean value of 10 accuracy values. This process was repeated 10 times, and the mean accuracy was obtained as the final classification accuracy. Kernel parameters were chosen through a 3-fold cross-validation approach, which divided the training dataset randomly into three subsets. The classifier was trained on either of the two subsets and tested on the third one. A set of parameters that provided the best cross-validation accuracy was employed for further analysis using the LIBSVM interface [71]. The kernel parameters with the best cross-validation accuracy were chosen to perform the actual classification. Under the conditions described above, weight values (*w*) and weight squared values (*w*^2^) of variables were obtained, with the latter ones ranging from large to small to form a variable weight table for further analysis. The MATLAB codes used to generate the results were provided in the Supplementary Materials.

All VIP > 1 variables filled their corresponding vacancies in the variable weight table. For those variables, their sequence in the weight table was sequential, implying that two methods, including VIP and weight ranking, to seek eligible variables have completely consistent findings. But for those variables, their VIP > 1 sequence in the weight table was not consecutive, implying some variables with VIP < 1 were also listed in the weight table. As mentioned above, SVMRFE has higher predictability than OPLS-DA in general, i.e., the weight ranking method is more reliable than the VIP method in picking eligible variables. Therefore, we still kept those variables with VIP < 1 in the weight table to prevent the possible loss of some valid variables during VIP computation for further analysis. Overlapped variables in 20 and 100 ng/mL groups, on behalf of their significantly low and high concentrations, underwent further verification by pairwise *t*-test [47,48,49] performed in SPSS Statistics software (Version 17.0) and fold change calculation of concentration during univariate analysis. Finally, eligible variables were confirmed to represent the marker compounds by comparing the retention time and precise molecular weight (absolute value of error less than 5 ppm) of 124 P&VDs (Table 2).

## 4. Conclusions

This study developed an SVM-assisted metabolomics method to screen the marker compounds of 124 P&VDs in maize samples. One hundred and twenty out of one hundred and twenty-four P&VDs can be identified by our proposed method, while only one hundred and nine P&VDs can be found by the metabolomics method alone, implying that SVM can promote the screening accuracy of the metabolomics method. It is the first time to apply the SVM-assisted metabolomics method to non-targeted screening of P&VDs in plant-derived foods. Method LODs were calculated for 120 P&VDs, with values significantly below 10 µg/kg for all of them, which were favorably comparable with a targeted multi-residue method. Our approach, developed on a simple and self-designed case, has been validated on a more complicated and realistic condition. This study promoted insight into the development of truly non-targeted screening approaches on the basis of metabolomics (particularly LC–MS/MS and chemometrics) for food safety assessment, which may be in need of non-targeted methods as a complement to targeted methods in view of screening potentially contaminated food products in the near future. Since our proposed method was rather generic, it was applicable with only a few modifications to any other LC–MS/MS dataset or even to other fields like authenticity or origin issues to complement existing methods. In conclusion, there are reasons to believe that these findings will encourage more breakthroughs in analytical issues, both in the methods and the tools.

## Figures and Tables

**Table 1:** Marker compounds were screened in maize sample groups.

No.	Var ID (Primary)	Marker Compounds	VIP Pred ^a^	(Weight Squared Value × 10^5^) ^b^	*m*/*z* ^c^	*t* (min) ^d^	Mass Error (ppm) ^e^	LOD (µg/kg)
**1**	M406T575	Difenoconazole	7.378/12.942	56,490/23,814	406.07130	9.58	−1.654	0.5
**2**	M326T524	Benalaxyl	5.798/5.975	9548/20,867	326.17516	8.73	0.279	1.1
**3**	M336T541	Zoxamide	4.658/5.732	11,861/11,744	336.03283	9.02	2.657	0.7
**4**	M218T216	Pymetrozine	7.828/5.649	25,040/13,579	218.10371	3.60	0.337	0.7
**5**	M372T574	Profenofos	6.189/5.531	26,626/4524	372.94268	9.57	0.699	0.9
**6**	M353T444	Tebufenozide	4.262/4.761	3052/4239	353.22383	7.40	4.178	1.0
**7**	M365T664	Pyridaben	0.514/4.492	73/3656	365.14408	11.07	−2.218	0.9
**8**	M256T210	Trichlorfon	2.470/4.263	689/3233	256.92941	3.50	−1.753	0.5
**9**	M218T300	Propanil	2.321/4.243	599/2611	218.01315	5.00	−1.160	1.3
**10**	M220T226	Dichlorvos	4.161/4.225	2493/2742	220.95367	3.77	2.220	0.9
**11**	M304T410	Fenpropimorph	3.241/4.191	1248/2663	304.26361	6.83	0.386	1.5
**12**	M223T241	Acetamiprid	4.561/4.170	3386/2965	223.07489	4.02	1.761	0.5
**13**	M349T621	Clorpyrifos	3.678/4.141	1708/2579	349.93393	10.35	1.049	0.5
**14**	M192T245	Carbendazim	2.651/4.094	746/2573	192.07637	4.08	−1.957	1.1
**15**	M732T421	Spinosad	4.227/4.059	2608/2462	732.46855	7.02	0.588	1.0
**16**	M294T344	Paclobutrazol	2.783/4.041	815/2289	294.13689	5.73	0.420	0.9
**17**	M345T650	Oxadiazon	3.066/3.997	1010/2326	345.07613	10.83	−1.724	1.0
**18**	M279T211	Oxadixyl	3.629/3.980	1621/2292	279.13363	3.52	−1.063	0.9
**19**	M276T326	Dimethenamid	3.496/3.968	1471/2338	276.08142	5.43	−1.904	0.8
**20**	M282T660	Pendimethalin	4.213/3.873	2396/2265	282.14480	11.00	−0.092	0.7
**21**	M305T541	Diazinon	4.289/3.844	2528/2250	305.10830	9.02	−0.114	0.4
**22**	M302T228	Flutriafol	4.602/3.813	3265/2173	302.11046	3.80	1.695	0.4
**23**	M330T429	Epoxiconazol	2.132/3.739	394/2123	330.08077	7.15	1.141	0.4
**24**	M324T499	Flutolanil	5.143/3.569	4610/1575	324.11992	8.32	−2.068	0.3
**25**	M292T384	Cyproconazol	3.236/3.488	1016/1409	292.12131	6.40	0.645	0.5
**26**	M253T239	Hexazinone	0.434/3.446	44/1392	253.16520	3.98	−2.774	0.8
**27**	M319T370	Pyriftalid	2.171/3.416	347/1241	319.07443	6.17	−0.885	0.9
**28**	M321T561	Chlorpyrifos-methyl	3.301/3.349	1028/1237	321.90281	9.35	1.702	1.1
**29**	M343T308	Thiophanate-methyl	1.896/3.345	249/1184	343.05204	5.13	−2.599	1.1
**30**	M306T540	Pirimiphos-methyl	7.863/3.343	24,423/1100	306.10351	9.00	−0.217	0.6
**31**	M888T502	Emamectin benzoate	1.008/3.257	106/1067	888.54603	8.37	−0.830	1.1
**32**	M337T436	Fenbuconazole	1.937/3.235	245/1010	337.12103	7.27	−1.260	0.9
**33**	M289T354	Myclobutanil	2.280/3.197	347/895	289.12143	5.90	−0.082	0.7
**34**	M224T228	Monocrotophos	4.047/3.145	1844/885	224.06760	3.80	−2.848	0.8
**35**	M222T196	Carbofuran	2.358/3.126	362/856	222.11230	3.27	−0.773	0.5
**36**	M304T200	Fenamiphos	1.974/3.103	237/790	304.11316	3.33	0.267	0.9
**37**	M299T446	Quinalphos	4.042/3.029	1806/729	299.06183	7.43	1.514	1.2
**38**	M338T555	Bitertanol	2.887/2.982	588/752	338.18671	9.25	1.223	0.9
**39**	M300T306	Phosphamidon	1.897/2.956	199/789	300.07668	5.10	1.536	0.8
**40**	M256T277	Phosfolan	1.855/2.946	188/728	256.02283	4.62	1.078	1.1
**41**	M271T540	Cadusafos	3.111/2.930	729/640	271.09408	9.00	−3.356	1.1
**42**	M376T359	Prochloraz	1.725/2.915	158/619	376.03841	5.98	0.850	0.5
**43**	M226T450	Cyprodinil	3.535/2.786	1080/608	226.13315	7.50	−3.196	0.5
**44**	M243T411	Mocap	1.673/2.746	132/591	243.06367	6.85	−0.080	0.9
**45**	M215T432	Metribuzin	2.828/2.720	507/396	215.09661	7.20	2.312	0.9
**46**	M436T523	Fipronil	4.356/2.698	2316/545	436.94589	8.72	−0.199	0.9
**47**	M250T234	Clothianidin	1.491/2.630	95/531	250.01693	3.90	3.708	0.9
**48**	M253T261	Thiacloprid	1.262/2.621	68/460	253.03037	4.35	−2.158	0.5
**49**	M292T214	Thiamethoxam	1.650/2.596	116/582	292.02655	3.57	−0.066	0.4
**50**	M294T360	Triadimefon	3.591/2.572	1107/393	294.10089	6.00	1.746	0.5
**51**	M302T266	Methidathion	3.877/2.538	1467/386	302.96913	4.43	−0.049	0.9
**52**	M368T509	Anilofos	1.299/2.491	65/377	368.03063	8.48	0.281	0.9
**53**	M299T346	Phoxim	1.463/2.430	79/367	299.06142	5.77	0.149	1.0
**54**	M318T521	Phosmet	0.495/2.374	31/326	318.00180	8.68	−0.050	0.6
**55**	M293T309	Etrimfos	2.431/2.362	279/281	293.07130	5.15	−2.200	0.8
**56**	M284T503	Metolachlor	1.320/2.289	57/312	284.14146	8.38	0.993	0.7
**57**	M330T488	Iprodione	1.610/2.279	88/299	330.04077	8.13	0.293	0.6
**58**	M216T346	Atrazine	2.591/2.276	334/284	216.10192	5.77	4.024	0.5
**59**	M307T499	Sulfotep	2.147/2.267	186/321	307.05231	8.32	−1.796	0.6
**60**	M208T297	Fenobucarb	1.281/2.247	51/261	208.13320	4.95	−0.059	1.0
**61**	M256T248	Imidacloprid	3.372/2.230	845/260	256.05943	4.13	−0.595	1.0
**62**	M203T178	Dinotefuran	2.636/2.201	345/241	203.11381	2.97	−0.305	1.1
**63**	M249T290	Linuron	2.276/2.189	521/222	249.01904	4.83	−0.689	0.8
**64**	M242T356	Prometryn	1.801/2.173	108/198	242.14414	5.93	3.079	0.5
**65**	M230T309	Terbutylazine	2.165/2.136	178/220	230.11703	5.15	1.443	0.8
**66**	M329T323	Pencycuron	1.550/2.124	71/221	329.14203	5.38	1.535	0.7
**67**	M318T276	Azinphos-methyl	1.632/1.675	60/94	318.01430	4.60	3.916	0.3
**68**	M279T490	Fenthion	0.372/1.438	8/54	279.02730	8.17	−0.006	0.9
**69**	M214T317	Simetryn	3.354/2.111	815/190	214.11264	5.28	2.581	0.5
**70**	M313T320	Praziquantel	1.624/2.045	76/183	313.19130	5.33	0.793	1.0
**71**	M479T347	Chlortetracycline	3.939/2.020	1530/182	479.12216	5.78	1.234	1.1
**72**	M463T377	Tetracycline	2.347/2.005	221/176	463.17183	6.28	1.560	0.3
**73**	M445T384	Doxycycline	3.592/1.990	1061/167	445.16071	6.40	0.390	0.5
**74**	M277T489	Sulfabenzamide	2.596/1.981	310/162	277.06427	8.15	0.462	0.4
**75**	M275T541	Ormetoprim	1.289/1.980	41/159	275.15083	9.02	2.093	0.6
**76**	M281T212	Sulfamonomethoxine	2.221/1.979	181/147	281.07014	3.53	−0.519	0.9
**77**	M315T378	Sulfaphenazole	0.518/1.908	28/144	315.09129	6.30	0.870	0.9
**78**	M215T306	Sulfacetamide	1.919/1.893	112/125	215.04915	5.10	3.056	0.9
**79**	M281T361	Sulfameter	2.102/1.822	142/112	281.07037	6.02	0.274	0.8
**80**	M279T225	Sulfamethazine	2.008/1.818	123/111	279.09161	3.75	2.104	0.9
**81**	M254T427	Sulfamethoxazole	1.564/1.815	58/118	254.05989	7.12	1.979	1.0
**82**	M265T308	Sulfamerazine	1.827/1.813	91/106	265.07593	5.13	2.102	0.8
**83**	M254T429	Sulfamethoxazole	0.616/1.811	16/105	254.05937	7.15	−0.063	0.8
**84**	M311T666	Sulfamethazine	1.671/1.800	71/103	311.08055	11.10	−0.962	0.8
**85**	M301T301	Sulfaquinoxaline	1.493/1.798	51/102	301.07589	5.02	1.739	1.0
**86**	M285T412	sulfachloropyridazine	1.001/1.758	19/95	285.02113	6.87	1.316	0.8
**87**	M215T293	Sulfaguanidine	1.926/1.734	103/97	215.05963	4.88	−0.403	0.9
**88**	M251T288	Sulfadiazine	2.213/1.692	164/96	251.05942	4.80	−1.177	0.4
**89**	M256T275	Sulfathiazole	2.800/1.687	396/99	256.02180	4.58	3.531	0.4
**90**	M279T377	Sulfisomidine	2.312/1.671	192/77	279.09146	6.28	1.584	0.4
**91**	M268T208	Sulfafurazole	1.659/1.668	64/87	268.07577	3.47	2.711	1.0
**92**	M291T365	Trimethoprim	1.707/1.652	70/77	291.14592	6.08	2.575	0.9
**93**	M281T380	Sulfamethoxypyridazine	0.735/1.636	26/89	281.07031	6.33	0.066	0.8
**94**	M360T414	Enrofloxacin	3.164/1.616	637/75	360.17198	6.90	0.491	0.8
**95**	M320T382	Norfloxacin	1.198/1.601	24/75	320.14194	6.37	4.507	0.7
**96**	M334T383	Pefloxacin	2.511/1.588	260/73	334.15581	6.38	−1.023	0.9
**97**	M332T404	Ciprofloxacin	1.473/1.573	37/70	332.14104	6.73	1.621	0.7
**98**	M362T385	Ofloxacin	1.266/1.535	27/68	362.15151	6.42	1.252	0.9
**99**	M386T456	Sarafloxacin	1.148/1.507	47/67	386.13203	7.60	2.492	0.4
**100**	M352T427	Lomefloxacin	0.728/1.451	7/62	352.14603	7.12	−1.973	0.3
**101**	M233T608	Nalidixic acid	1.036/1.449	15/56	233.09243	10.13	1.528	0.3
**102**	M262T619	Flumequine	2.101/1.441	128/55	262.08760	10.32	0.770	0.5
**103**	M358T411	Danofloxacin	0.814/1.415	7/52	358.15716	6.85	2.823	0.8
**104**	M400T442	Difloxacin	1.830/1.414	77/50	400.14683	7.37	0.282	0.8
**105**	M396T433	Orbifloxacin	1.500/1.395	38/48	396.15371	7.22	1.927	1.0
**106**	M393T494	Sparfloxacin	0.671/1.393	10/48	393.17268	8.23	−1.498	0.9
**107**	M734T616	Erythromycin	1.106/1.365	16/46	734.46808	10.27	−0.599	0.9
**108**	M837T659	Roxithromycin	1.236/1.354	23/38	837.53167	10.98	−0.214	1.0
**109**	M828T630	Josamycin	1.349/1.351	68/36	828.47461	10.50	0.733	0.8
**110**	M916T611	Tylosin	1.039/1.348	13/36	916.52689	10.18	0.505	0.9
**111**	M702T622	Kitasamycin	1.758/1.345	65/36	702.40593	10.37	0.010	0.8
**112**	M869T566	Tilmicosin	0.491/1.343	7/35	869.57374	9.43	0.484	1.0
**113**	M128T254	2-methyl-5-nitroimidazole	2.095/1.336	269/35	128.04551	4.23	0.444	0.9
**114**	M114T265	4-nitroimidazole	1.036/1.333	14/32	114.02989	4.42	0.821	1.0
**115**	M164T246	5-nitrobenzimidazole	1.122/1.319	16/32	164.04613	4.10	4.115	1.3
**116**	M142T207	Dimetridazole	1.492/1.311	36/32	142.06163	3.45	3.755	0.5
**117**	M172T218	Metronidazole	2.251/1.281	164/31	172.07180	3.63	0.779	1.1
**118**	M201T240	Ronidazole	1.356/1.280	26/31	201.06230	4.00	2.315	0.4
**119**	M158T329	Hydroxy dimetridazole	2.126/1.262	131/31	158.05646	5.48	2.797	0.4
**120**	M170T362	Ipronidazole	1.305/1.258	23/31	170.09277	6.03	2.156	0.9

Note: ^a^ two VIP values from 20 vs. 50 and 100 and 100 vs. 20 and 50, respectively; ^b^ two weight squared values (×10^5^) from 20 vs. 50 and 100 and 100 vs. 20 and 50, respectively; ^c^ *m*/*z* represents extracted molecular weight from W4M platform; ^d^ *t* represents retention time from W4M platform; ^e^ Mass error (ppm) = (extracted molecular weight from W4M platform − extracted molecular weight from LC–MS/MS) × 10^6^/extracted molecular weight from LC–MS/MS.

**Table 2:** Basic information on 69 pesticides (No. **1**~**69**) and 55 veterinary drugs (No. **70**~**124**).

No.	Compounds	Category	CAS No.	Extracted Molecular Weight	Retention Time (min)
**1**	Dinotefuran	insecticide	165252-70-0	203.11387	3.06
**2**	Carbofuran	insecticide	1563-66-2	222.11247	3.21
**3**	Fenamiphos	insecticide	22224-92-6	304.11308	3.44
**4**	Trichlorfon	insecticide	52-68-6	256.92986	3.47
**5**	Thiamethoxam	insecticide	153719-23-4	292.02657	3.54
**6**	Pymetrozine	insecticide	123312-89-0	218.10364	3.61
**7**	Dichlorvos	insecticide	62-73-7	220.95318	3.78
**8**	Monocrotophos	insecticide	2157-98-4	224.06824	3.81
**9**	Clothianidin	insecticide	210880-92-5	250.01600	3.95
**10**	Acetamiprid	insecticide	135410-20-7	223.07450	4.03
**11**	Imidacloprid	insecticide	105827-78-9	256.05958	4.09
**12**	Thiacloprid	insecticide	111988-49-9	253.03092	4.33
**13**	Methidathion	insecticide	950-37-8	302.96914	4.37
**14**	Phosfolan	insecticide	947-02-4	256.02255	4.54
**15**	Azinphos-methyl	insecticide	86-50-0	318.01305	4.59
**16**	Fenobucarb	insecticide	3766-81-2	208.13321	4.96
**17**	Phosphamidon	insecticide	13171-21-6	300.07622	5.05
**18**	Etrimfos	insecticide	38260-54-7	293.07194	5.16
**19**	Phoxim	insecticide	14816-18-3	299.06138	5.71
**20**	Mocap	insecticide	13194-48-4	243.06369	6.89
**21**	Spinosad	insecticide	131929-60-7	732.46812	7.15
**22**	Tebufenozide	insecticide	112410-23-8	353.22235	7.35
**23**	Quinalphos	insecticide	13593-03-8	299.06138	7.41
**24**	Fenthion	insecticide	55-38-9	279.02730	8.07
**25**	Sulfotep	insecticide	3689-24-5	307.05286	8.26
**26**	Emamectin benzoate	insecticide	155569-91-8	888.54677	8.54
**27**	Phosmet	insecticide	732-11-6	318.00182	8.57
**28**	Fipronil	insecticide	120068-37-3	436.94598	8.73
**29**	Cadusafos	insecticide	95465-99-9	271.09499	8.95
**30**	Diazinon	insecticide	333-41-5	305.10833	8.96
**31**	Pirimiphos-methyl	insecticide	29232-93-7	306.10358	8.96
**32**	Chlorpyrifos-methyl	insecticide	5598-13-0	321.90226	9.32
**33**	Profenofos	insecticide	41198-08-7	372.94242	9.49
**34**	Clorpyrifos	insecticide	2921-88-2	349.93356	10.26
**35**	Pyridaben	insecticide	96489-71-3	365.14489	11.06
**36**	Oxadixyl	bactericide	77732-09-3	279.13393	3.49
**37**	Flutriafol	bactericide	76674-21-0	302.10995	3.79
**38**	Carbendazim	bactericide	10605-21-7	192.07675	4.11
**39**	Thiophanate-methyl	bactericide	23564-05-8	343.05293	5.14
**40**	Pencycuron	bactericide	66063-05-6	329.14152	5.47
**41**	Prochloraz	bactericide	67747-09-5	376.03809	5.89
**42**	Myclobutanil	bactericide	88671-89-0	289.12145	6.06
**43**	Triadimefon	bactericide	43121-43-3	294.10038	6.11
**44**	Cyproconazol	bactericide	113096-99-4	292.12112	6.36
**45**	Fenpropimorph	bactericide	67306-03-0	304.26349	6.72
**46**	Epoxiconazol	bactericide	106325-08-0	330.08039	7.11
**47**	Fenbuconazole	bactericide	114369-43-6	337.12145	7.33
**48**	Cyprodinil	bactericide	121552-61-2	226.13387	7.64
**49**	Iprodione	bactericide	36734-19-7	330.04067	8.13
**50**	Flutolanil	bactericide	66332-96-5	324.12059	8.23
**51**	Benalaxyl	bactericide	71626-11-4	326.17507	8.75
**52**	Zoxamide	bactericide	156052-68-5	336.03194	8.99
**53**	Bitertanol	bactericide	55179-31-2	338.18630	9.17
**54**	Difenoconazole	bactericide	119446-68-3	406.07197	9.64
**55**	Hexazinone	herbicide	51235-04-2	253.16590	3.94
**56**	Linuron	herbicide	330-55-2	249.01921	4.92
**57**	Propanil	herbicide	709-98-8	218.01340	4.98
**58**	Terbutylazine	herbicide	5915-41-3	230.11670	5.07
**59**	Simetryn	herbicide	1014-70-6	214.11209	5.32
**60**	Dimethenamid	herbicide	87674-68-8	276.08195	5.41
**61**	Atrazine	herbicide	102029-43-6	216.10105	5.77
**62**	Prometryn	herbicide	7287-19-6	242.14339	5.97
**63**	Pyriftalid	herbicide	135186-78-6	319.07471	6.02
**64**	Metribuzin	herbicide	21087-64-9	215.09611	7.14
**65**	Metolachlor	herbicide	51218-45-2	284.14118	8.34
**66**	Anilofos	herbicide	64249-01-0	368.03053	8.49
**67**	Oxadiazon	herbicide	19666-30-9	345.07672	10.77
**68**	Pendimethalin	herbicide	40487-42-1	282.14483	10.92
**69**	Paclobutrazol	growth regulator	76738-62-0	294.13677	5.77
**70**	Sulfafurazole	sulfamido	127-69-5	268.07504	3.38
**71**	Sulfamonomethoxine	sulfamido	1220-83-3	281.07029	3.54
**72**	Sulfamethazine	sulfamido	57-68-1	279.09102	3.76
**73**	Sulfathiazole	sulfamido	72-14-0	256.02090	4.54
**74**	Sulfadiazine	sulfamido	68-35-9	251.05972	4.73
**75**	Sulfaguanidine	sulfamido	57-67-0	215.05972	4.85
**76**	Sulfaquinoxaline	sulfamido	59-40-5	301.07537	5.05
**77**	Sulfacetamide	sulfamido	144-80-9	215.04849	5.06
**78**	Sulfamerazine	sulfamido	127-79-7	265.07537	5.11
**79**	Sulfameter	sulfamido	651-06-9	281.07029	5.95
**80**	Trimethoprim	sulfamido	738-70-5	291.14517	6.01
**81**	Sulfaphenazole	sulfamido	526-08-9	315.09102	6.21
**82**	Sulfisomidine	sulfamido	515-64-0	279.09102	6.24
**83**	Sulfamethoxypyridazine	sulfamido	80-35-3	281.07029	6.51
**84**	Sulfachloropyridazine	sulfamido	80-32-0	285.02075	6.87
**85**	Sulfamethoxazole	sulfamido	723-46-6	254.05939	7.09
**86**	Sulfamethoxazole	sulfamido	723-46-6	254.05939	7.09
**87**	Sulfabenzamide	sulfamido	127-71-9	277.06414	8.05
**88**	Ormetoprim	sulfamido	6981-18-6	275.15025	8.94
**89**	Sulfamethazine	sulfamido	57-68-1	311.08085	11.05
**90**	Dimetridazole	nitroimidazoles	551-92-8	142.06110	3.46
**91**	Metronidazole	nitroimidazoles	443-48-1	172.07167	3.58
**92**	Ronidazole	nitroimidazoles	7681-76-7	201.06183	3.96
**93**	5-nitrobenzimidazole	nitroimidazoles	94-52-0	164.04545	4.05
**94**	2-methyl-5-nitroimidazole	nitroimidazoles	88054-22-2	128.04545	4.17
**95**	4-nitroimidazole	nitroimidazoles	3034-38-6	114.02980	4.41
**96**	Hydroxy dimetridazole	nitroimidazoles	936-05-0	158.05602	5.55
**97**	Ipronidazole	nitroimidazoles	14885-29-1	170.09240	6.05
**98**	Mebendazole	nitroimidazoles	31431-39-7	296.10297	7.74
**99**	Fleroxacin	quinolones	79660-72-3	370.13730	5.99
**100**	Ofloxacin	quinolones	82419-36-1	362.15106	6.39
**101**	Pefloxacin	quinolones	70458-92-3	334.15615	6.41
**102**	Norfloxacin	quinolones	70458-96-7	320.14050	6.51
**103**	Ciprofloxacin	quinolones	85721-33-1	332.14050	6.73
**104**	Enrofloxacin	quinolones	93106-60-6	360.17180	7.01
**105**	Danofloxacin	quinolones	112398-08-0	358.15615	7.02
**106**	Lomefloxacin	quinolones	98079-51-7	352.14672	7.12
**107**	Orbifloxacin	quinolones	113617-63-3	396.15295	7.27
**108**	Difloxacin	quinolones	98106-17-3	400.14672	7.42
**109**	Sarafloxacin	quinolones	98105-99-8	386.13107	7.64
**110**	Sparfloxacin	quinolones	110871-86-8	393.17327	8.34
**111**	Nalidixic acid	quinolones	389-08-2	233.09207	10.01
**112**	Flumequine	quinolones	42835-25-6	262.08740	10.15
**113**	Praziquantel	vermifuge	55268-74-1	313.19105	5.31
**114**	Chlortetracycline	tetracyclines	57-62-5	479.12157	5.68
**115**	Tetracycline	tetracyclines	60-54-8	463.17111	6.21
**116**	Doxycycline	tetracyclines	564-25-0	445.16054	6.34
**117**	Lincomycin	macrolides	154-21-2	407.22104	9.41
**118**	Tilmicosin	macrolides	108050-54-0	869.57332	9.49
**119**	Clindamycin	macrolides	18323-44-9	425.18715	9.68
**120**	Tylosin	macrolides	1401-69-0	916.52643	10.23
**121**	Erythromycin	macrolides	114-07-8	734.46852	10.25
**122**	Kitasamycin	macrolides	1392-21-8	702.40592	10.48
**123**	Josamycin	macrolides	16846-24-5	828.47400	10.65
**124**	Roxithromycin	macrolides	80214-83-1	837.53185	10.84
**125**	Atrazine-d5		163165-75-1	221.14017	5.77
**126**	Enrofloxacin-d5		1173021-92-5	365.21092	7.01

## Data Availability

The data presented in this study are available in this article and Supplementary Materials.

## Supplementary Materials

The following supporting information can be downloaded at: https://www.mdpi.com/article/10.3390/molecules29133026/s1, MATLAB codes to run SVM; Table S1: Recovery of internal standards in maize sample groups; Table S2: Pairwise *t*-test results of 154 variables; Table S3: Fold change results of 154 variables; Table S4: Concentration of P&VDs in maize samples from Jinpu New Area. Figure S1. Cluster analysis plot of spiked maize sample groups.
