# Supplementary material for: A Support Vector Machine-Assisted Metabolomics Approach for Non-Targeted Screening of Multi-Class Pesticides and Veterinary Drugs in Maize
Source: Molecules. 2024 Jun 26;29(13):3026. doi: 10.3390/molecules29133026 (PMC11243018; doi:10.3390/molecules29133026)
Supplement: Supplementary file 1 [file molecules-29-03026-s001.zip › molecules-3024607-supplementary.pdf]

Supplementary Materials

MATLAB codes to run SVM

```
load data.mat;
load label.mat;
[data_normal] = premnmx(data);
k =10;
sum_accuracy_svm = 0;
[m,n] = size(data_normal);
indices = crossvalind('Kfold',m,k);
for i = 1:k
test_indic = (indices == i);
train_indic = ~test_indic;
train_data = data_normal(train_indic,:);
train_label = label(train_indic,:);
test_data = data_normal(test_indic,:);
test_label = label(test_indic,:);
end;
classifer = fitcsvm(train_data,train_label);
predict_label  = predict(classifer, test_data);
accuracy_svm = length(find(predict_label == test_labels))/length(test_label);
sum_accuracy_svm = sum_accuracy_svm + accuracy_svm;
mean_accuracy_svm = sum_accuracy_svm/k;
disp(mean_accuracy_svm);
n = size(data,2);
s = 1:n;
r = [];
iter = 1;
while ~isempty(s);
if mod(iter, 10) == 0;
str = ['===',num2str(iter),'==='];
disp(str);
disp('processing .....');
end;
X = data(:,s);
v = 3;
[bestCVaccuracy,bestc,bestg] = SVMcgForClass(label, X, -8,8,-8,8,v,0.8,0.8,4.5);
```

```
cmd = ['-c ',num2str(bestc),' -g ',num2str(bestg)];
model = svmtrain(label, X, cmd);
model = svmtrain(label, X);
w = model.SVs' * model.sv_coef;
c = w.^2;
[c_minvalue, f] = min(c);
r = [s(f),r];
ind = [1:f-1, f+1:length(s)];
s = s(ind);
iter = iter + 1;
end
```

Table S1. Recovery (%) of internal standards in maize sample groups (n=9).

Item	Concentration groups (ng/mL)	1	2	3	4	5	6	7	8	9	RSD (%)
Recovery (%) of enrofloxacin-d5	20	77.4	80.2	93.1	83.2	85.7	92.7	75.6	88.1	89.7	7.5
	50	72.9	91.2	90.7	78.2	86.7	93.4	87.5	86.9	90.1	7.7
	100	77.7	93.6	82.4	74.9	74.9	91.3	80.1	88.6	86.7	8.4
Recovery (%) of atrazine-d5	20	95.2	78.3	91.6	90.3	73.6	84.7	92.7	88.1	83.2	8.2
	50	94.7	81.2	93.6	88.7	82.6	91.2	88.2	91.7	90.0	5.2
	100	96.4	87.4	93.4	87.3	72.9	84.2	88.6	77.5	86.0	8.4
Final recovery (%)	20	85.4	79.2	92.3	86.6	79.2	88.5	83.3	88.1	86.3	5.0
	50	82.4	85.9	92.1	83.1	84.6	92.3	87.8	89.2	90.0	4.2
	100	86.0	90.4	87.6	80.6	73.9	87.6	84.1	82.7	86.3	5.8

Table S2. Pairwise t-test results of 154 variables.

Var ID (Primary)	p_{20vs50}	$p_{20vs100}$	$p_{50vs100}$	Var ID (Primary)	p_{20vs50}	$p_{20vs100}$	$p_{50vs100}$	Var ID (Primary)	p_{20vs50}	$p_{20vs100}$	$p_{50vs100}$
M406T575	0.000	0.000	0.000	M299T346	0.000	0.000	0.000	M396T433	0.000	0.000	0.000
M326T524	0.000	0.000	0.000	M318T521	0.000	0.000	0.000	M393T494	0.000	0.000	0.000
M218T216	0.000	0.000	0.000	M307T499	0.000	0.000	0.000	M429T764	0.000	0.000	0.000
M336T541	0.000	0.000	0.000	M284T503	0.000	0.000	0.000	M734T616	0.000	0.000	0.000
M372T574	0.000	0.000	0.000	M330T488	0.000	0.000	0.000	M332T404	0.000	0.000	0.000
M353T444	0.000	0.000	0.000	M216T346	0.000	0.000	0.000	M396T433	0.000	0.000	0.000
M365T664	0.000	0.000	0.000	M293T309	0.000	0.000	0.000	M837T659	0.000	0.000	0.000
M256T210	0.000	0.000	0.000	M208T297	0.000	0.000	0.000	M828T630	0.000	0.000	0.000
M223T241	0.000	0.000	0.000	M256T248	0.000	0.000	0.000	M916T611	0.000	0.000	0.000
M220T226	0.000	0.000	0.000	M203T178	0.000	0.000	0.000	M702T622	0.000	0.000	0.000
M304T410	0.000	0.000	0.000	M249T290	0.000	0.000	0.000	M869T566	0.000	0.000	0.000
M218T300	0.000	0.000	0.000	M329T323	0.000	0.000	0.000	M128T254	0.000	0.000	0.000
M349T621	0.000	0.000	0.000	M230T309	0.000	0.000	0.000	M114T265	0.000	0.000	0.000
M192T245	0.000	0.000	0.000	M242T356	0.000	0.000	0.000	M164T246	0.000	0.000	0.000
M732T421	0.000	0.000	0.000	M214T317	0.000	0.000	0.000	M142T207	0.000	0.000	0.000
M276T326	0.000	0.000	0.000	M313T320	0.000	0.000	0.000	M393T495	0.000	0.000	0.000
M345T650	0.000	0.000	0.000	M479T347	0.000	0.000	0.000	M172T218	0.000	0.000	0.000
M279T211	0.000	0.000	0.000	M463T377	0.000	0.000	0.000	M201T240	0.000	0.000	0.000
M294T344	0.000	0.000	0.000	M445T384	0.000	0.000	0.000	M158T329	0.000	0.000	0.000
M282T660	0.000	0.000	0.000	M277T489	0.000	0.000	0.000	M170T362	0.000	0.000	0.000
M305T541	0.000	0.000	0.000	M275T541	0.000	0.000	0.000	M284T581	0.000	0.000	0.000
M302T228	0.000	0.000	0.000	M281T212	0.000	0.000	0.000	M138T814	0.000	0.000	0.000
M330T429	0.000	0.000	0.000	M315T378	0.000	0.000	0.000	M495T677	0.000	0.000	0.000
M324T499	0.000	0.000	0.000	M215T306	0.000	0.000	0.000	M222T383	0.000	0.000	0.000
M292T384	0.000	0.000	0.000	M254T427	0.000	0.000	0.000	M108T707	0.000	0.000	0.000
M253T239	0.000	0.000	0.000	M281T361	0.000	0.000	0.000	M165T577	0.000	0.000	0.000
M319T370	0.000	0.000	0.000	M279T225	0.000	0.000	0.000	M133T417	0.000	0.000	0.000
M321T561	0.000	0.000	0.000	M265T308	0.000	0.000	0.000	M167T857	0.000	0.000	0.000

M343T308	0.000	0.000	0.000	M254T429	0.000	0.000	0.000	M442T663	0.000	0.000	0.000
M306T540	0.000	0.000	0.000	M311T666	0.000	0.000	0.000	M241T50_2	0.000	0.000	0.000
M888T502	0.000	0.000	0.000	M301T301	0.000	0.000	0.000	M178T774	0.000	0.000	0.000
M337T436	0.000	0.000	0.000	M256T275	0.000	0.000	0.000	M185T572	0.000	0.000	0.000
M289T354	0.000	0.000	0.000	M215T293	0.000	0.000	0.000	M108T832	0.000	0.000	0.000
M224T228	0.000	0.000	0.000	M251T288	0.000	0.000	0.000	M237T120	0.000	0.000	0.000
M222T196	0.000	0.000	0.000	M285T412	0.000	0.000	0.000	M179T50	0.000	0.000	0.000
M304T200	0.000	0.000	0.000	M318T276	0.000	0.000	0.000	M120T93	0.000	0.000	0.000
M300T306	0.000	0.000	0.000	M281T380	0.000	0.000	0.000	M138T475	0.000	0.000	0.000
M338T555	0.000	0.000	0.000	M268T208	0.000	0.000	0.000	M280T222	0.000	0.000	0.000
M299T446	0.000	0.000	0.000	M291T365	0.000	0.000	0.000	M158T441	0.000	0.000	0.000
M256T277	0.000	0.000	0.000	M279T377	0.000	0.000	0.000	M196T88	0.000	0.000	0.000
M271T540	0.000	0.000	0.000	M360T414	0.000	0.000	0.000	M234T601	0.000	0.000	0.000
M376T359	0.000	0.000	0.000	M320T382	0.000	0.000	0.000	M181T57	0.000	0.000	0.000
M226T450	0.000	0.000	0.000	M334T383	0.000	0.000	0.000	M302T836	0.000	0.000	0.000
M243T411	0.000	0.000	0.000	M332T404	0.000	0.000	0.000	M184T266	0.000	0.000	0.000
M292T214	0.000	0.000	0.000	M362T385	0.000	0.000	0.000	M165T458	0.000	0.000	0.000
M436T523	0.000	0.000	0.000	M386T456	0.000	0.000	0.000	M178T274	0.000	0.000	0.000
M250T234	0.000	0.000	0.000	M352T427	0.000	0.000	0.000	M147T401	0.000	0.000	0.000
M253T261	0.000	0.000	0.000	M233T608	0.000	0.000	0.000	M295T814	0.000	0.000	0.000
M215T432	0.000	0.000	0.000	M262T619	0.000	0.000	0.000	M311T388	0.000	0.000	0.000
M294T360	0.000	0.000	0.000	M279T490	0.000	0.000	0.000	M422T537	0.000	0.000	0.000
M302T266	0.000	0.000	0.000	M358T411	0.000	0.000	0.000				
M368T509	0.000	0.000	0.000	M400T442	0.000	0.000	0.000				

p_{20vs50}, $p_{20vs100}$ and $p_{50vs100}$ represent the significance level of variables between the two concentration groups.

Table S3. Fold change (FC) results of 154 variables.

Var ID (Primary)	FC_{50vs20}	$FC_{100vs20}$	Var ID (Primary)	FC_{50vs20}	$FC_{100vs20}$	Var ID (Primary)	FC_{50vs20}	$FC_{100vs20}$
M406T575	2.415	5.244	M299T346	2.543	5.211	M396T433	2.604	4.776
M326T524	2.466	4.876	M318T521	2.611	5.098	M393T494	2.549	4.783
M218T216	2.378	4.788	M307T499	2.356	5.192	M429T764	2.445	5.091
M336T541	2.668	4.562	M284T503	2.433	4.999	M734T616	2.621	4.981
M372T574	2.354	4.674	M330T488	2.554	4.912	M332T404	2.551	4.893
M353T444	2.601	4.890	M216T346	2.455	5.002	M396T433	2.523	4.904
M365T664	2.440	5.201	M293T309	2.335	5.089	M837T659	2.514	4.990
M256T210	2.542	4.671	M208T297	2.610	5.043	M828T630	2.390	5.000
M223T241	2.631	4.893	M256T248	2.561	5.079	M916T611	2.348	5.090
M220T226	2.444	4.672	M203T178	2.544	4.891	M702T622	2.435	5.212
M304T410	2.741	5.332	M249T290	2.511	4.990	M869T566	2.455	4.723
M218T300	2.542	5.129	M329T323	2.542	4.732	M128T254	2.671	4.784
M349T621	2.633	5.332	M230T309	2.509	4.900	M114T265	2.517	4.788
M192T245	2.589	5.013	M242T356	2.449	5.120	M164T246	2.498	5.128
M732T421	2.341	5.125	M214T317	2.658	5.099	M142T207	2.554	5.125
M276T326	2.655	5.222	M313T320	2.498	5.339	M393T495	2.334	5.221
M345T650	2.444	4.891	M479T347	2.344	4.700	M172T218	2.413	4.976
M279T211	2.433	5.011	M463T377	2.431	4.902	M201T240	2.599	4.788
M294T344	2.576	4.786	M445T384	2.512	5.101	M158T329	2.572	4.901
M282T660	2.601	4.903	M277T489	2.509	5.119	M170T362	2.442	4.912
M305T541	2.343	4.672	M275T541	2.514	5.112	M284T581	2.501	4.782
M302T228	2.611	4.781	M281T212	2.337	5.214	M138T814	2.400	4.891
M330T429	2.548	4.991	M315T378	2.441	4.881	M495T677	2.391	4.901
M324T499	2.445	4.789	M215T306	2.456	4.678	M222T383	2.372	4.733
M292T384	2.487	5.234	M254T427	2.465	4.901	M108T707	2.490	4.567
M253T239	2.550	5.011	M281T361	2.374	4.983	M165T577	2.516	4.556
M319T370	2.551	5.123	M279T225	2.511	4.782	M133T417	2.561	4.691
M321T561	2.451	5.099	M265T308	2.490	4.671	M167T857	2.451	5.091
M343T308	2.490	5.213	M254T429	2.398	4.981	M442T663	2.671	5.099
M306T540	2.501	4.899	M311T666	2.441	4.777	M241T50_2	2.661	5.145
M888T502	2.431	4.893	M301T301	2.561	4.893	M178T774	2.541	5.221
M337T436	2.376	4.981	M256T275	2.544	5.124	M185T572	2.561	4.898
M289T354	2.433	5.001	M215T293	2.612	4.783	M108T832	2.549	4.782
M224T228	2.541	5.110	M251T288	2.338	5.019	M237T120	2.333	5.211
M222T196	2.496	4.789	M285T412	2.459	5.112	M179T50	2.443	4.983

M304T200	2.458	4.982	M318T276	2.661	4.894	M120T93	2.341	5.212
M300T306	2.509	5.134	M281T380	2.431	4.789	M138T475	2.540	4.881
M338T555	2.551	5.098	M268T208	2.572	5.019	M280T222	2.442	4.678
M299T446	2.512	4.789	M291T365	2.561	5.178	M158T441	2.413	5.215
M256T277	2.509	4.555	M279T377	2.498	4.993	M196T88	2.409	5.009
M271T540	2.389	5.221	M360T414	2.490	4.894	M234T601	2.451	4.999
M376T359	2.430	4.761	M320T382	2.371	5.120	M181T57	2.471	5.004
M226T450	2.451	5.223	M334T383	2.441	4.893	M302T836	2.453	4.712
M243T411	2.544	4.764	M332T404	2.612	4.981	M184T266	2.496	4.891
M292T214	2.398	4.798	M362T385	2.561	4.783	M165T458	2.500	4.742
M436T523	2.477	5.231	M386T456	2.511	5.086	M178T274	2.335	5.092
M250T234	2.651	4.897	M352T427	2.413	4.900	M147T401	2.604	4.902
M253T261	2.335	5.112	M233T608	2.432	4.891	M295T814	2.556	5.144
M215T432	2.514	4.563	M262T619	2.445	5.112	M311T388	2.543	5.123
M294T360	2.499	4.782	M279T490	2.449	5.171	M422T537	2.612	4.908
M302T266	2.389	4.780	M358T411	2.378	5.211			
M368T509	2.345	5.013	M400T442	2.421	4.989			

FC_{50vs20} and $FC_{100vs20}$ represent the fold change of peak intensity of variables between the two concentration groups.

Table S4. Concentration (μg/kg) of P&VDs in maize samples (*n*=7) from Jinpu New Area.

Compounds	Sampling sites	
	Qili Village	Bali Village
Norfloxacin	10.7±0.2	11.7±0.3
Enrofloxacin	11.9±0.3	11.3±0.3
Imidacloprid	17.7±0.3	14.2±0.2
Carbendazim	14.7±0.3	12.4±0.3
Total concentration	55.0	49.6

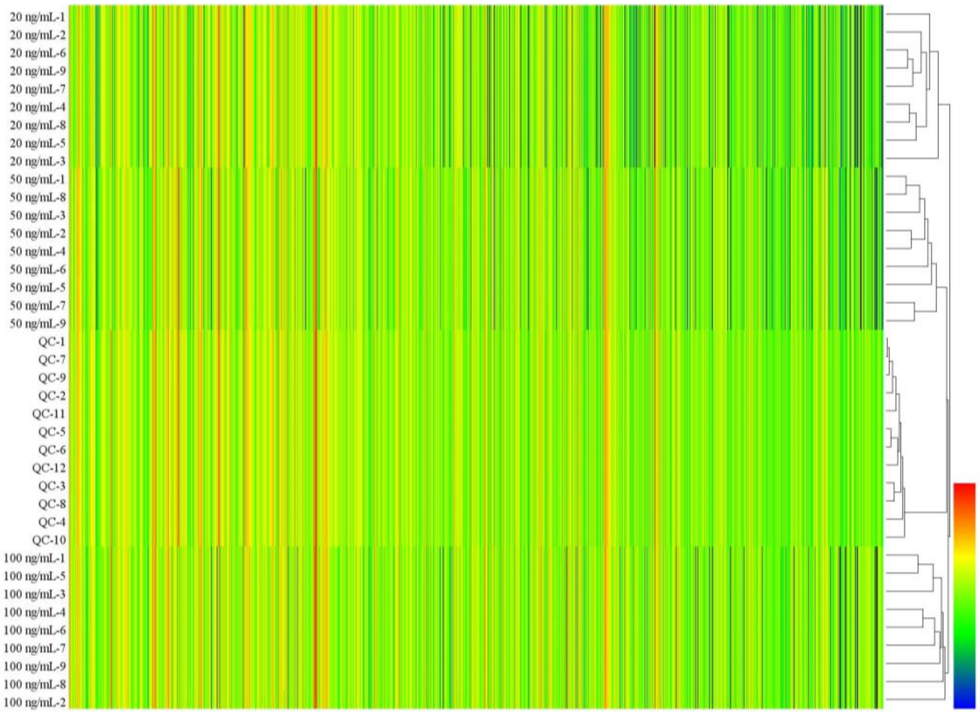

Figure S1. Cluster analysis plot of spiked maize sample groups.
